# Turns and downturns in aging drivers

**DOI:** 10.1016/j.trip.2026.102214

**Published:** 2026-08-26

**Authors:** Marie Hardt, Guillermo Basulto-Elias, Heike Hofmann, Shauna Hallmark, Anuj Sharma, Jeffrey D. Dawson, Matthew Rizzo, Jun Ha Chang

**Affiliations:** a Department of Statistics, Iowa State University, 2438 Osborn Dr., Ames, IA 50011, USA; b Institute for Transportation, Iowa State University, 2711 S. Loop Drive Suite 4700, Ames, IA 50010, USA; c Department of Statistics, University of Nebraska-Lincoln, 340 Hardin Hall North Wing, Lincoln, NE 68583, USA; d Department of Biostatistics, University of Iowa, 145 N. Riverside Dr., Iowa City, IA 52242, USA; e Department of Neurological Sciences, University of Nebraska Medical Center, 4242 Farnam St. Suite 650, Omaha, NE 68131, USA

**Keywords:** Older drivers, Cognitive decline, Naturalistic driving study, Left-turning behavior, Digital behavioural markers, Mild cognitive impairment, Alzheimer’sdisease

## Abstract

As cognitive decline progresses, older adults may self-regulate their driving. Avoidance of left turns across traffic is observable in naturalistic driving data but rarely self-reported. We studied 106 older adults using baseline and one-year follow-up neuropsychological assessments. In-vehicle sensors passively recorded driving behavior over 12 weeks. We identified 216,531 turns from vehicle heading changes. We used mixed-effects logistic regression to model the odds of turning left, with cognitive status category change from baseline to one-year follow-up as the predictor. Greater cognitive impairment, represented by movement to a more severe cognitive status category at one-year follow-up, was associated with reduced odds of turning left (odds ratio = 0.98, 95 % confidence interval = (0.964, 0.996); P value = 0.011). Left-turn avoidance may be a behavioral marker of early cognitive decline. Passive driving data could help detect functional changes earlier, enabling intervention to preserve mobility and independence.

## 1. Background

Early detection of cognitive decline, particularly mild cognitive impairment (MCI) and Alzheimer’s disease (AD), is a critical priority for clinical care and public health. While neuropsychological testing remains the gold standard, there is growing interest in identifying real-world behavioral biomarkers that reflect subtle, prodromal changes. Driving—a cognitively demanding activity involving attention, visuospatial processing, executive function, and motor coordination—offers a natural context to observe such changes.

Naturalistic driving data allow for the passive capture of decision-making and motorist behavior in everyday settings without experimental direction (Howell et al. 2024). In a review of the literature available as of 2022, Bayat and Roe noted that several naturalistic driving studies involving older drivers, where data was collected by the Driving Real-world In-Vehicle Evaluation System (DRIVES), showed that older drivers with preclinical AD drove less often than their cognitively normal counterparts and were also less likely to adopt aggressive driving behaviors like hard braking, speeding, and sudden acceleration (Bayat and Roe 2022). Intersections, in particular, pose significant challenges for older drivers due to their high cognitive and perceptual demands. Age-related cognitive decline increases crash risk, especially during complex maneuvers like left turns (Basulto-Elias et al. 2023).

Older drivers are disproportionately involved in intersection crashes. For example, those aged 65–69 are 2.3 times more likely and those aged 85+ are 10.6 times more likely than younger drivers to be involved in a fatal intersection crash (Preusser et al. 1998). Naturalistic driving studies such as that conducted under the Second Strategic Highway Research Program (SHRP 2) have confirmed an elevated intersection crash risk in drivers over 70 (Seacrist et al. 2020). The risk continues to rise with age and is further amplified in drivers with cognitive disorders. A recent meta-analysis identified intersection navigation as one of the most common sources of errors in drivers with dementia (Camilleri and Whitehead 2023).

Left turns are particularly hazardous. In focus-group interviews of eight older South Korean drivers, Choi et al. (2025) asked the study participants about driving situations where they felt stressed, and left turns were identified as a stressful driving situation. Older drivers are frequently cited for failure to yield, especially at stop-controlled intersections, where older drivers are significantly more likely than younger drivers to be at fault in a crash (Sifrit et al. 2011; Braitman et al. 2007). Failure-to-yield errors often occur during left turns and are more common among drivers 70 and older (FHWA 1995).

Some older drivers compensate for perceived deficits by avoiding nighttime driving or high-traffic environments. However, avoidance of left turns, a cognitively and perceptually demanding maneuver, is rarely self-reported (Molnar and Eby 2008; Molnar et al. 2013; Beck et al. 2022), despite being observable in driving data.

A recent study from the Longitudinal Research on Aging Drivers (LongROAD) project showed that the monthly ratio of right turns to left turns was among the most predictive features in classifying drivers with MCI or dementia (Di et al. 2023). This suggests that passive, sensor-based monitoring of turning behavior can capture subtle behavioral changes not reflected in self-reporting.

While the LongROAD study provided important insights, it relied on cross-sectional classification based on the most severe cognitive state and excluded participants with shorter study participation. It also did not distinguish between MCI, dementia, and AD.

Because driving involves complex, goal-directed behavior, early-stage cognitive dysfunction may manifest as changes in turning behavior long before a formal diagnosis is made. Identifying these changes supports the development of behavioral biomarkers for cognitive impairment. Such biomarkers provide indications of when older drivers should undergo cognitive evaluations and whether driving cessation is necessary for the protection of the driver and other roadway users. Additionally, understanding the driving challenges faced by older adults informs roadway design and traffic control strategies to enhance safety.

This study evaluates whether older drivers with cognitive decline exhibit reduced odds of making left turns. It brings together interdisciplinary contributions from statisticians, transportation researchers, and neurologists. By comparing turning behaviors among older drivers with and without cognitive decline, we assess the usefulness of left-turn frequency as a real-world marker of early cognitive change. Earlier detection of age-related cognitive decline allows measures to preserve mobility and independence to be taken sooner, extending quality of life. Knowledge about an older driver’s cognitive status plays a role in decisions about driving cessation, which is an essential safety consideration.

## 2. Methods

### 2.1. Study design and data collection

This study used interim data from an ongoing naturalistic driving study sponsored by the University of Nebraska Medical Center (UNMC). The parent study was designed to include a baseline cognitive assessment and the collection of three months of real-world driving behavior using in-vehicle sensors and wearable devices, followed by annual cognitive assessments over a two-year period. At the time of this analysis, data from the baseline and one-year follow-up assessments were available. The UNMC Institutional Review Board approved the protocol (0522–20-FB), and informed consent was obtained from all participants.

Each participant’s vehicle was equipped with custom-built sensor instrumentation packages called Black Boxes developed by Digital Artefacts LLC that passively recorded data from when the vehicle ignition was turned on to when it was turned off. The vehicle Black Boxes were identical to those used by Joshi et al. (2025). Each ignition cycle (on to off) defined a single “drive.” The system included forward-facing roadway and cabin cameras, an Adafruit #790 Global Positioning System (GPS) module with an integrated antenna (GPS accuracy < 3 m and velocity accuracy 0.1 m/s), and a Bosch Sensortec BNO055 9-DOF inertial measurement unit (IMU) incorporating an accelerometer, a gyroscope, a magnetometer (heading accuracy up to ±2.5°), and a microcontroller for sensor fusion. On-board diagnostic (OBD) data were recorded via an OBDLink MX WiFi Scan Tool, but were not utilized in this analysis. The GPS module operated at 5 Hz and the IMU operated at approximately 100 Hz. Downsampling was performed to create the 1 Hz data used for this analysis.

We note that standardized road tests and driving simulators are frequently used to assess driver performance. However, these data sources utilize identical real-world driving courses or virtual driving scenarios. We would then expect that each study participant would complete the same number of left and right turns as they navigated the course, whether in the real world or in a simulator. Since these driving scenarios are controlled and practically the same for each study participant, there would be no value in analyzing the number of left and right turns taken by the participants in these scenarios. In contrast, naturalistic driving studies permit participants to drive as they normally would. The drivers make choices about their routes (whether consciously or unconsciously) and thus make an analysis of turning behavior meaningful.

### 2.2. Participants

We enrolled 182 active, legally licensed drivers aged 65 to 90 years from the Omaha, Nebraska area. Participants were recruited through social media, newspapers, flyers, and outreach to community organizations (Merickel et al. 2019).

The same inclusion criteria as Chang et al. (2026) were used. Briefly, all participants met Nebraska’s visual acuity standards (better than 20/50 OU, corrected or uncorrected) and self-reported no exclusionary medical conditions (e.g., dementia, heart failure, psychiatric illness) or use of psychoactive medications. Common age-related conditions like arthritis were not exclusionary. Participants completed baseline and one-year follow-up questionnaires on demographics, driving experience, medical history, and socioeconomic status. Table 1 summarizes the participants’ characteristics and the turning endpoints.

Upon enrollment, each participant’s baseline cognitive status was assessed. The cognitive assessment criteria are discussed in Section 2.3.2.

### 2.3. Measures

#### 2.3.1. Turn detection

We identified turning behavior using gyroscopic heading (yaw) data, which are more reliable than GPS data at the low speeds typical of intersections. The raw gyroscopic heading was calculated by sensor-fusing gyroscope and magnetometer (compass) data from the 9-DOF IMU. As a post-processing step, the raw gyroscopic heading data were then downsampled to a frequency of 1 Hz.

The gyroscopic heading was then continuity-corrected to simplify subsequent calculations. The angle differences in gyroscopic heading between each point and the previous point were found. For the initial gyroscopic heading for each drive, the angle difference was set to 0. For each point, the cumulative sum of changes in gyroscopic heading up to and including that point was added to the initial gyroscopic heading value to create the continuity-corrected gyroscopic heading. The continuity-corrected gyroscopic heading modulus 360 is equivalent to the original gyroscopic heading.

Turning maneuvers are associated with increasing (for right turns) and decreasing (for left turns) patterns in continuity-corrected gyroscopic heading. Thus, a right turn corresponds to a period when the derivative of the continuity-corrected gyroscopic heading is positive. Similarly, a left turn corresponds to a period when the derivative of the continuity-corrected gyroscopic heading is negative. We use the centered difference approximation to find the first derivative for the continuity-corrected gyroscopic heading. The centered difference approximation to the first derivative is given in Equation (1) below:

(1) $$f(x)\approx \frac{f(x+h)-f(x-h)}{2h},$$

where h>0. In our case, h=1 since the data points are sampled with a frequency of 1 Hz (1 observation per second).

Next, we identified sign changes in the first derivative approximation. The sign may be positive, negative, or zero. For each interval where the first derivative approximation has the same sign, we subtracted the first continuity-corrected gyroscopic heading for the interval from the last continuity-corrected gyroscopic heading to quantify the amount of gyroscopic heading change within the interval.

Lastly, we defined a threshold *θ* for the change in continuity-corrected gyroscopic heading over an interval. A turn candidate was a right turn if the change in continuity-corrected gyroscopic heading over the interval was greater than *θ*. A turn candidate was a left turn if the change in continuity-corrected gyroscopic heading over the interval was less than −*θ*. The choice of *θ* impacted the extent to which non-turning behaviors, such as proceeding straight through a roundabout and driving on a curved road, were classified as turns.

We performed a sensitivity analysis to choose the threshold *θ* for our turn detection method and assess its impact on the mixed-effects logistic regression model described in Section 3.1.2. Our intuition suggested that for a typical turn at an intersection, the change in gyroscopic heading should be around ±90°, with the sign depending on whether it is a left (negative) or right (positive) turn. However, gyroscopic heading changes corresponding to turns in real-world driving scenarios may not reach ±90°. Thus, we explored values of *θ* ranging from ±30° to ±100° in increments of ±5°. The results of the sensitivity analysis are given in Table A.1 in the Appendix. The mixed-effects logistic regression model coefficient estimate and p-value were stable for reasonable choices of *θ* between ±50° and ±80°. We chose a turn detection threshold of *θ* = ±70°.

Fig. 1 illustrates the turn detection process for a right turn. The top panel shows the raw gyroscopic heading. Because the driver was going north, the gyroscopic headings jump between 0 and 360 degrees. The second panel shows how the continuity correction smooths out the jumps in the gyroscopic heading. The third panel shows the first derivative of the continuity-corrected gyroscopic heading. The gray shaded region shows where a right turn was detected with a threshold of *θ* = 70° for the change in gyroscopic heading over the interval where the first derivative was positive.

#### 2.3.2. Cognitive status classification

Cognitive status was assessed through a baseline assessment and a one-year follow-up assessment using standardized neurological and neuropsychological evaluations (Albert et al. 2011), which included domains such as memory, language, visuospatial skills, executive function, and attention. The criteria used were similar to those used by Chang et al. (2025). Classification into categories for normal cognition, MCI, or dementia was based on National Institute on Aging – Alzheimer’s Association (NIA-AA) and National Alzheimer’s Coordinating Center (NACC) Uniform Data Set (UDS) version 3 criteria, including the Petersen/Winblad and Jak/Bondi definitions (Petersen et al. 1999; Jak et al. 2009).

Functional assessments used the Clinical Dementia Rating (CDR) and Functional Activities Questionnaire (FAQ) (Marshall et al., 2015; Morris, 1993). For participants with MCI, the global CDR was expected to be 0.5 and the FAQ < 9. An FAQ > 9 and a CDR ≥ 0.5 indicated functional decline in alignment with dementia. Two clinicians with expertise in dementia reviewed the full battery of neuropsychological tests, functional assessments (CDR and FAQ), and medical and medication history to reach a consensus on cognitive status classification for each participant. The same assessment and evaluation criteria were used at baseline and follow-up.

Several criteria were used to exclude participants from the model. We excluded N = 50 participants from the model because they had not yet completed the one-year follow-up cognitive assessment. Participants were also excluded if they showed cognitive reversion (N = 14) at the one-year follow-up assessment or dementia at the baseline assessment (N = 7), which focused the analysis on normal and MCI trajectories. As shown in Fig. 2, the majority of participants who did not exhibit cognitive reversion had a period of up to 15 months between the end of the driving data collection period and the one-year follow-up cognitive assessment. To ensure similarity in the amount of time from the end of the driving data collection period to the one-year follow-up cognitive assessment, N = 4 participants were excluded because they had more than 15 months from the end of driving data collection to the one-year follow-up assessment. Another participant who had only 61 days between the end of the driving period and the one-year follow-up assessment was also excluded because these dates were much closer than for any other participant. This participant is not shown in Fig. 2. A total of 106 participants were included in the model. Final classifications included four cognitive trajectory groups based on baseline and follow-up status:

Normal→Normal(stable normal)

Normal→MCI(incident MCI)MCI→MCI(stable MCI)MCI→AD(progression to dementia)

## 3. Statistical analyses

To evaluate the relationship between turning behavior and cognitive prognosis, we used a mixed-effects logistic regression model, discussed in Section 3.1.

### 3.1. Mixed-effects logistic regression model

#### 3.1.1. Outcome and predictor variables for the logistic regression model

The dependent variable for the logistic regression model was a binary indicator of turn direction: 1 for left turns, 0 for right turns. The independent variable was the cognitive prognosis class (1 through 4), treated as a numeric ordinal variable reflecting increasing severity of cognitive decline.

#### 3.1.2. Logistic regression model specification and justification

We used mixed-effects logistic regression to model the odds of a driver making a left versus a right turn, with participant-level random intercepts to account for repeated measures. The model associates concurrent decline with past driving behavior. We specify the turn model (Equation (2)) as:

(2) $$\mathrm{logit}(P(Y_{ij}=1))=\beta_{0}+\beta_{1}X_{i1}+\gamma_{i},$$

where $Y_{ij}$ denotes the turn outcome (1 = left, 0 = right) for detected turn j made by study participant i, $\beta_{0}$ is a fixed intercept, $X_{i1}$ is the cognitive prognosis class for study participant i and can have a value of 1, 2, 3, or 4, as described in Section 2.3.2, $\beta_{1}$ is a fixed effect associated with the cognitive prognosis, and $\gamma_{i}$ is a participant-level random effect. We assume that the random effect $\gamma_{i}$ is normally distributed with mean 0 and variance $\sigma_{\gamma }^{2}$.

Note that, in this model, each turn is included as a separate event. This means that there is an implicit weighting for more active drivers. While there are similar trends at the participant level, we do not have enough participants to meet the threshold of significant differences. Even with a small overall number of study participants and small cognitive prognosis class sizes, the overall average of around 1000 left and right turns (combined) detected per study participant provides a large number of observations and lends power to the model. Fig. 3 shows a histogram of the total number of turns (left and right) we detected for each participant. Based on this, we do not have concerns that drivers with more or fewer detected turns will disproportionately influence the model estimates.

We tested the assumption that cognitive prognosis could be treated as a numeric ordinal variable (rather than a categorical variable) using a likelihood ratio test (Williams 2020). The test was performed using the R function anova. The test statistic, *χ*^2^ = 3.687, df = 2, P value = 0.158, supported the simpler ordinal model.

Random intercepts accounted for between-subject variation; thus, we did not adjust for age, sex, or driving experience, but rather assumed that these were captured by subject-level variation. The model was fit in R using the glmer function from the package lmerTest (Kuznetsova et al. 2017).

#### 3.1.3. Mixed-effects logistic regression model results

##### 3.1.3.1. Participants.

Among the 106 study participants included in the model, our algorithm identified 216,531 turns: 109,904 were classified as left turns and 106,627 as right turns. Participant demographic characteristics and turning endpoints are presented in Table 1. Note that SD denotes the standard deviation and IQR denotes the interquartile range.

The histogram in Fig. 4 shows the overall distribution of weekly driving distance (in miles). Most study participants did not drive very far, often less than 100 miles per week.

Fig. 5 shows boxplots of the percentage of left turns by cognitive prognosis group. The orange intervals represent 95 % bootstrap confidence intervals for the mean (Efron and Tibshirani 1993). A decreasing trend in the percentage of left turns is observed with increasing severity of cognitive impairment. This is clearly seen in the median percentage of left turns for each cognitive prognosis class, denoted by the black vertical line inside each box. Variability also differs across the four cognitive prognosis classes, as indicated by the different lengths of the boxes and whiskers for each cognitive prognosis class. The boxplots clearly show a relationship between cognitive prognosis class and turning frequency.

##### 3.1.3.2. Mixed-effects logistic regression model estimates.

The estimate of $\beta_{1}$ (the fixed effect associated with cognitive prognosis class) is −0.02 with a standard error of 0.008. This leads to an odds ratio (and effect size) estimate of 0.98 with a 95 % confidence interval of (0.964, 0.996) and a P value of 0.011. Thus, each unit increase in cognitive prognosis class (indicating the driver’s progression to more severe cognitive impairment) is associated with a 0.98-fold decrease in the predicted odds of the driver making a left turn. Equivalently, such an increase in the driver’s cognitive prognosis class is associated with a 1.02-fold increase in the predicted odds of the driver making a right turn. The estimate of $\sigma_{\gamma }^{2}$ (the variance of the participant-level random effect) is 0.005. The model results suggest that as cognitive impairment progresses over time, older drivers become less likely to make left turns.

We estimated predicted probabilities of turning left (i.e., marginal effects) for each cognitive prognosis class using the function predict_response from the R package ggeffects (Lüdecke 2018). The predicted probabilities of turning left and their corresponding 95 % confidence intervals are given in Table 2. The predicted probability of turning left decreases across cognitive prognosis classes.

## 4. Discussion

### 4.1. Conclusions

This study investigated the relationship between cognitive status and the likelihood of making left turns among older drivers. Using naturalistic driving data and longitudinal cognitive assessments, we found that greater cognitive impairment was significantly associated with reduced odds a driver making a left turn. Conversely, a lower frequency of left turns was associated with greater cognitive decline.

Unlike prior research relying on self-reported avoidance of certain driving situations (e.g., Molnar and Eby, (2008); Molnar et al. (2013); Beck et al., (2022)), our approach utilized passively collected real-world driving behavior. Consistent with Di et al. (2023), who identified the ratio of right turns to left turns as a predictive feature of cognitive impairment, our findings reinforce the value of turning behavior as a digital behavioral marker. Our longitudinal model strengthens this evidence by linking turn frequency to clinically verified cognitive trajectories. Importantly, our findings are based on changes observed over just one year. A longer-term longitudinal study using a second annual follow-up assessment is underway to determine whether these trends persist or evolve over time.

There are medical procedures to detect the presence of AD pathology in the brain, such as medical imaging or a physically invasive lumbar puncture. However, Bayat et al. (2021) observed that the utility of such diagnostics is limited by availability, cost, and older adults’ willingness to undergo them. Naturalistic driving data collection is undemanding on the driver, with the cost of the privacy loss associated with GPS location tracking. The ethics and practicality of such passive monitoring are beyond the scope of this article.

Our findings demonstrate the usefulness of passive driving data in monitoring for cognitive decline in older drivers. Our turn-detection approach used heading data from vehicle trajectories. Technologies such as smartphone apps make this data type fairly simple to collect, in contrast to approaches such as that of Basulto-Elias et al. (2023), which required the installation of in-vehicle video cameras to record driver glances. A longer driving data collection period or constant monitoring of turning behavior allows a baseline to be established for individual drivers. Deviations from baseline may then be noted and a clinical threshold for concern established for decreasing trends in left turn frequency. Our approach then provides an early warning that a cognitive evaluation is necessary. Detecting age-related cognitive decline earlier allows intervention to preserve mobility and independence for older drivers. It also provides valuable evidence for decisions about driving cessation, promoting safety for older adults and other roadway users.

### 4.2. Limitations

This study has several limitations. First, the study cohort consisted of primarily white, community-dwelling drivers aged 65 to 90 from the Omaha, Nebraska area. This may restrict the generalizability of the analysis results to other older drivers of different demographics living in other areas.

Second, our heading-based method for detecting turns was simple and broad and may have included non-intersection maneuvers, such as navigating road curves or lane changes. Future work should explore more precise turn detection approaches. Our methods also did not account for physical factors like intersection design. However, the large number of turns detected per study participant gives a stable estimate of each participant’s turning behavior in different scenarios throughout the driving data collection period.

Third, this study included a total of 106 participants, with group sizes ranging from 11 to 43, well below the sample sizes suggested by Babulal et al. of about 150 members per group to identify meaningful behavioral differences between older adults with and without preclinical AD (Babulal et al. 2019). By including each participant’s turns, we gain the necessary power to show the significant relationship between cognitive prognosis and turning behavior at the population level. However, this structurally prevents us from including any other potentially important subject-level covariates, such as age, sex, or driving experience. This is because the change in mental cognition was only observed once before and once after the driving period. There are clearly strong subject-to-subject differences in driving behavior, mitigated by the inclusion of the participant-level random effect.

The fact that a relationship between driving behavior and cognitive prognosis can be observed at a population level indicates that future studies should explore the use of individual baselines and subsequent follow-up monitoring of changes in driving behavior. Multiple driving periods or continuous monitoring of the driving behavior would allow an analysis with more comprehensive models that incorporate multiple covariates and utilize more advanced methods, such as random forests, to enhance predictive accuracy (Rizzo and Dawson, 2025).

## Figures and Tables

**Fig. 1.**
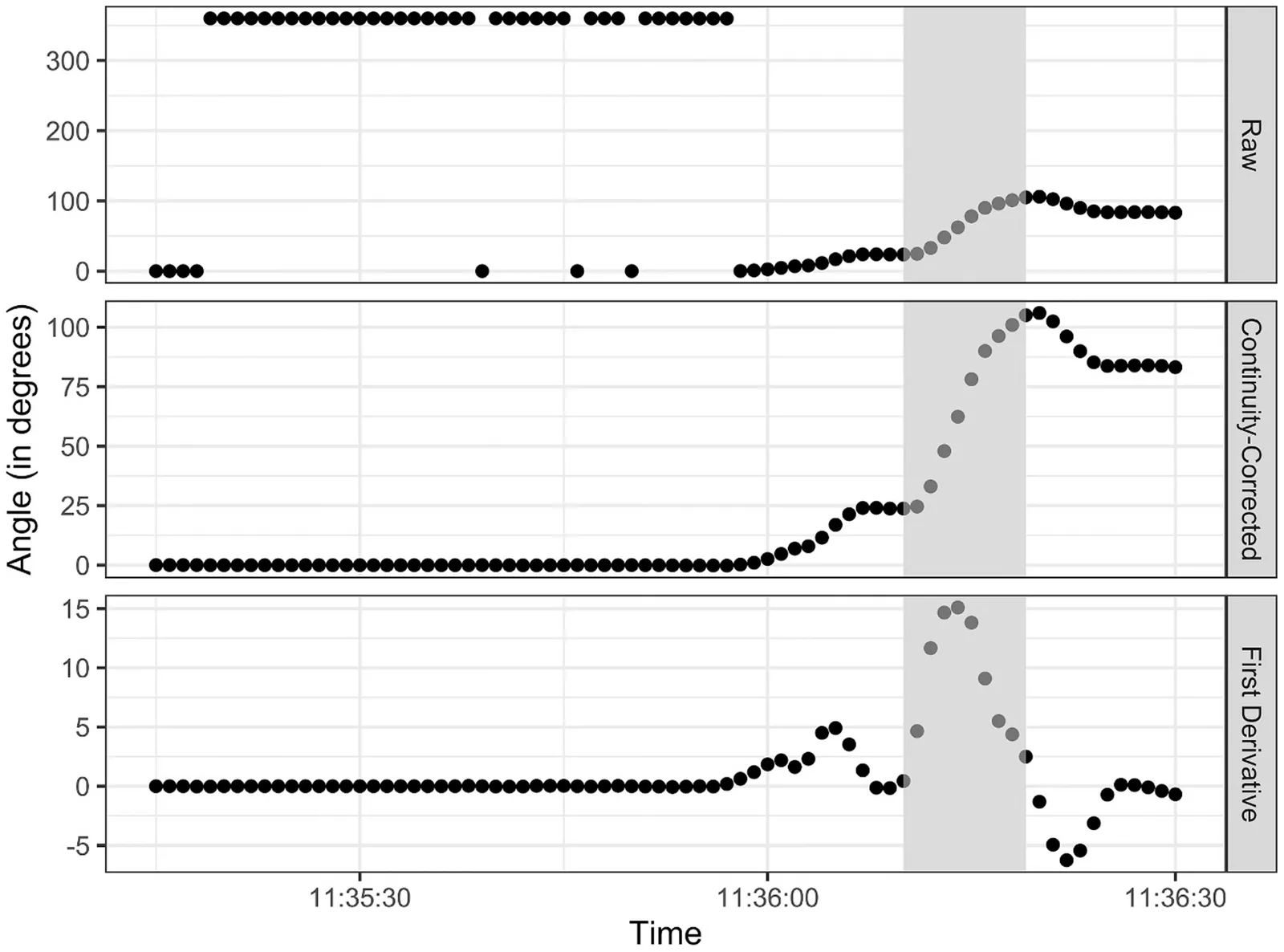
The turn detection process for a right turn. The raw gyroscopic heading is in the first panel, the continuity-corrected gyroscopic heading is in the second panel, and the first derivative of the continuity-corrected gyroscopic heading is in the third panel. The gray shaded region shows where a right turn was detected.

**Fig. 2.**
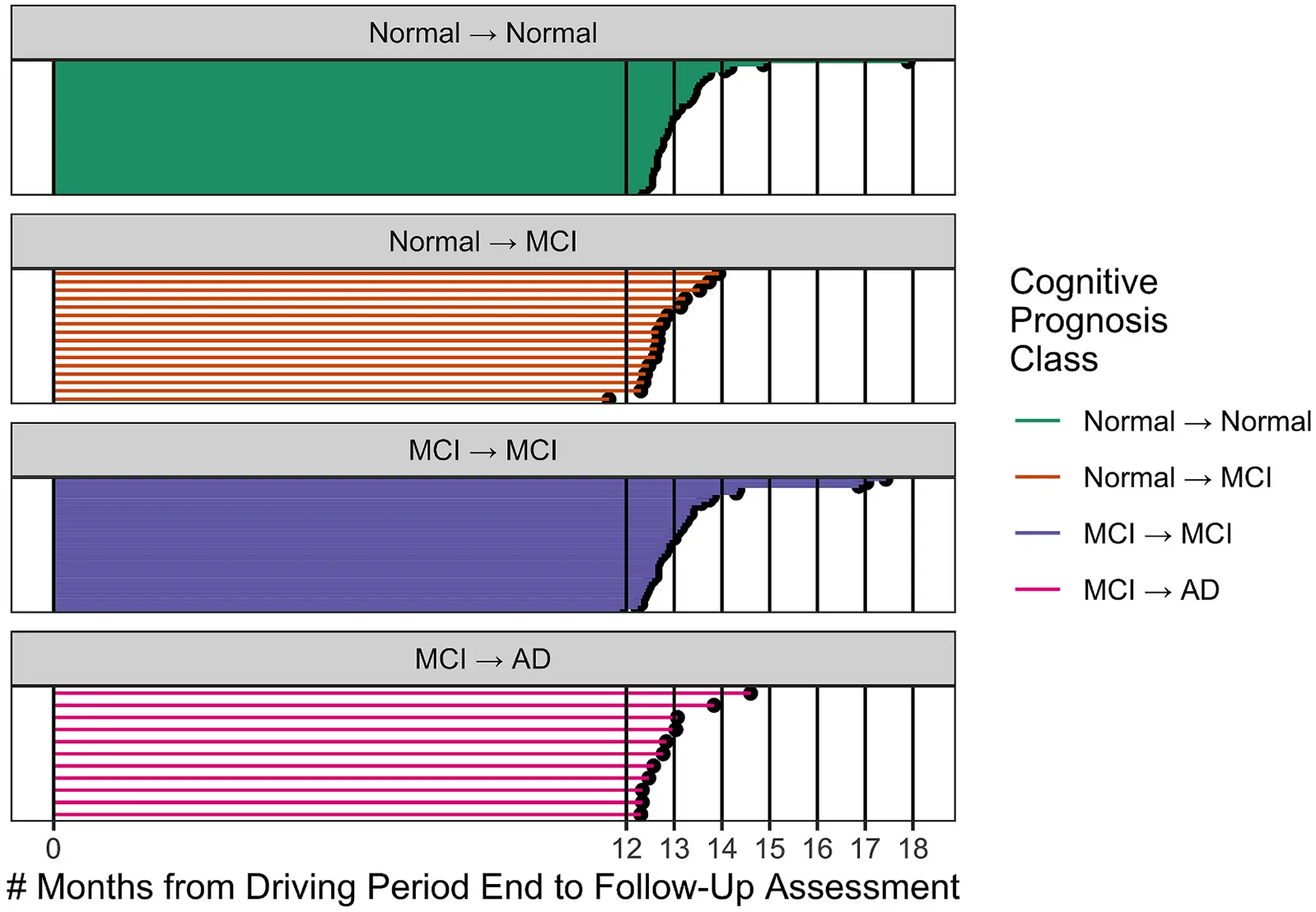
The number of months between the end of the driving data collection period and the follow-up cognitive assessment. Using a cutoff of 15 months, 4 participants were excluded from the model.

**Fig. 3.**
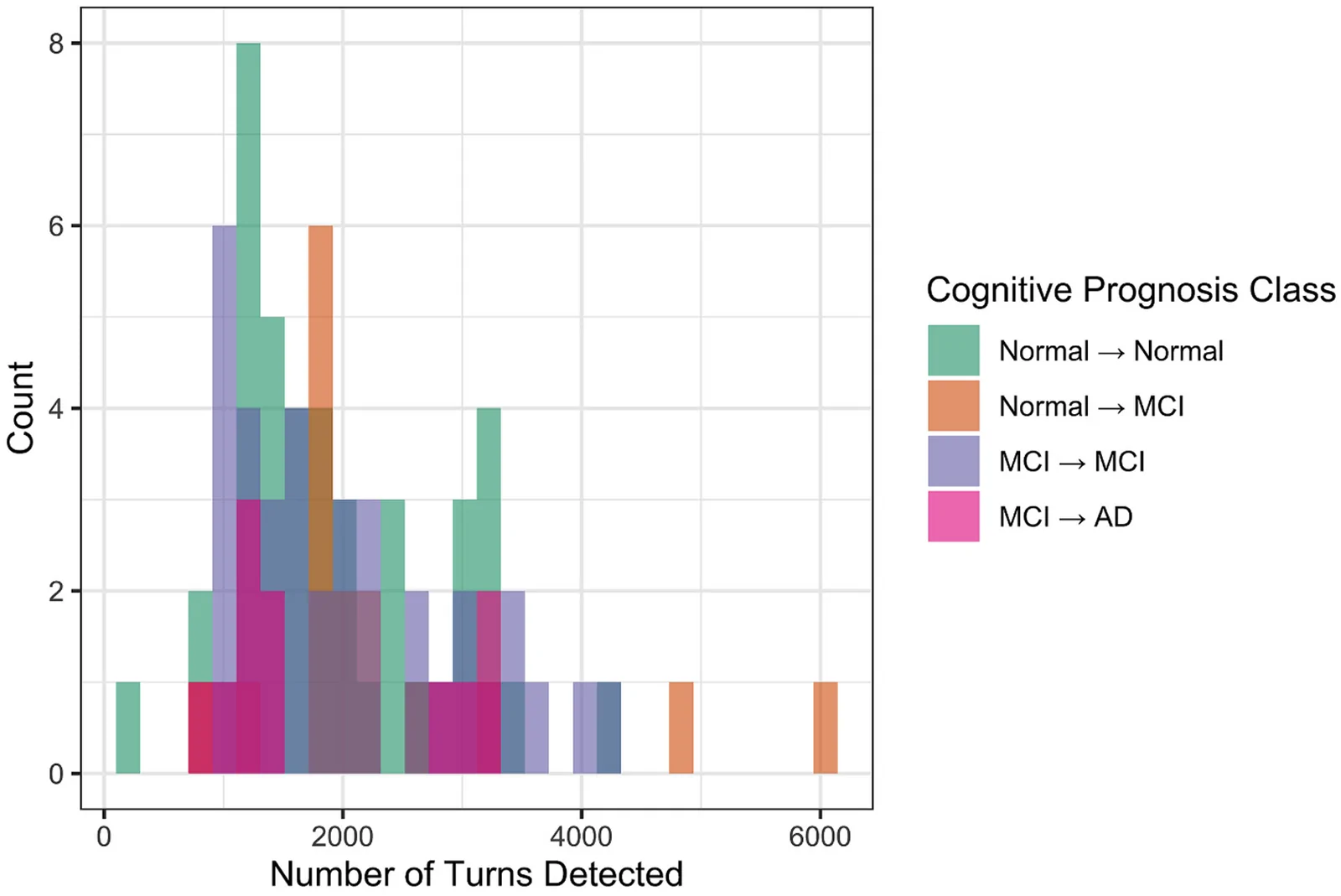
Histogram of the total number of turns (left and right) detected for each participant in the four cognitive prognosis classes. The majority of participants had a similar number of detected turns.

**Fig. 4.**
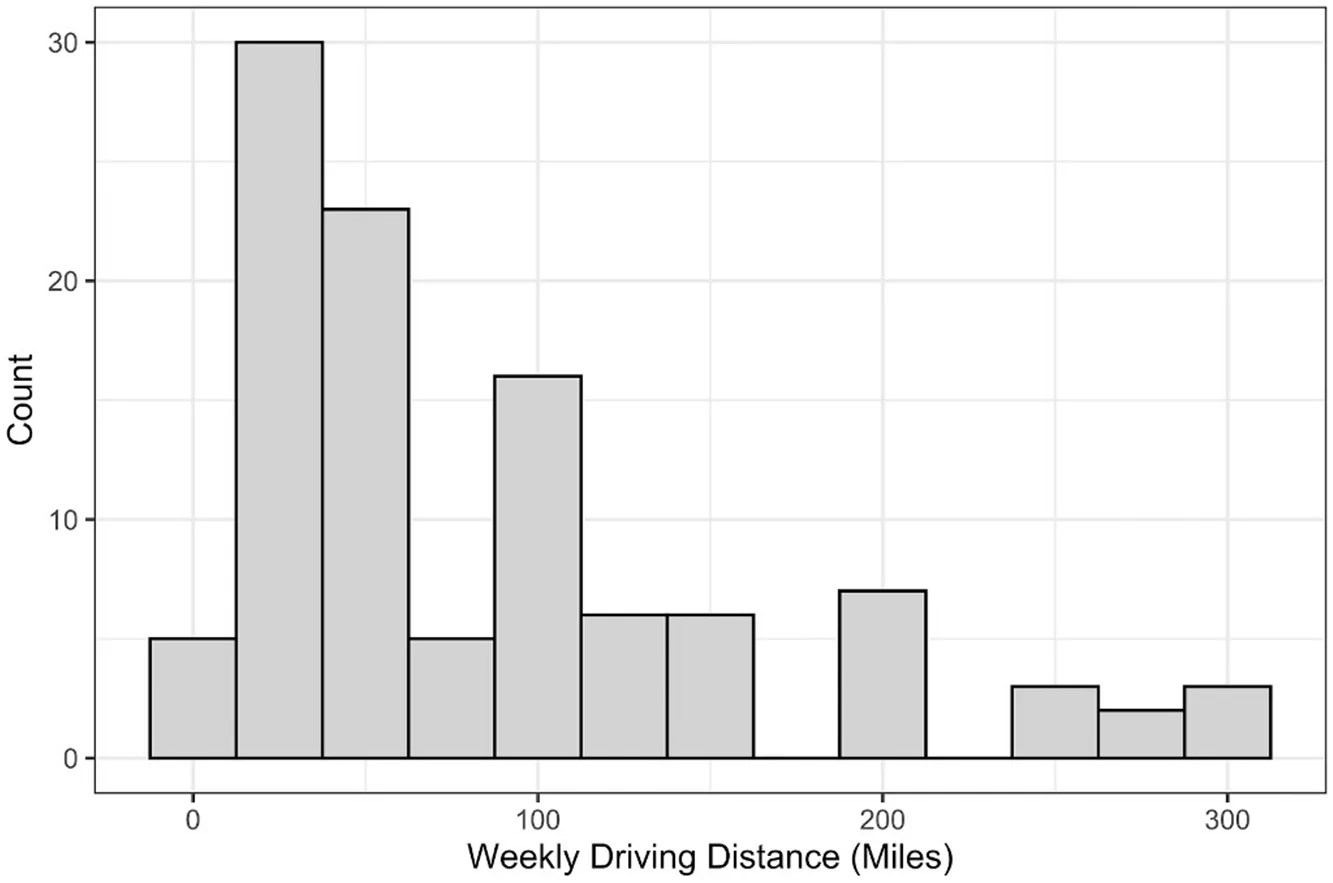
Histogram of Weekly Driving Distance (Miles). Most study participants drove less than 100 miles per week.

**Fig. 5.**
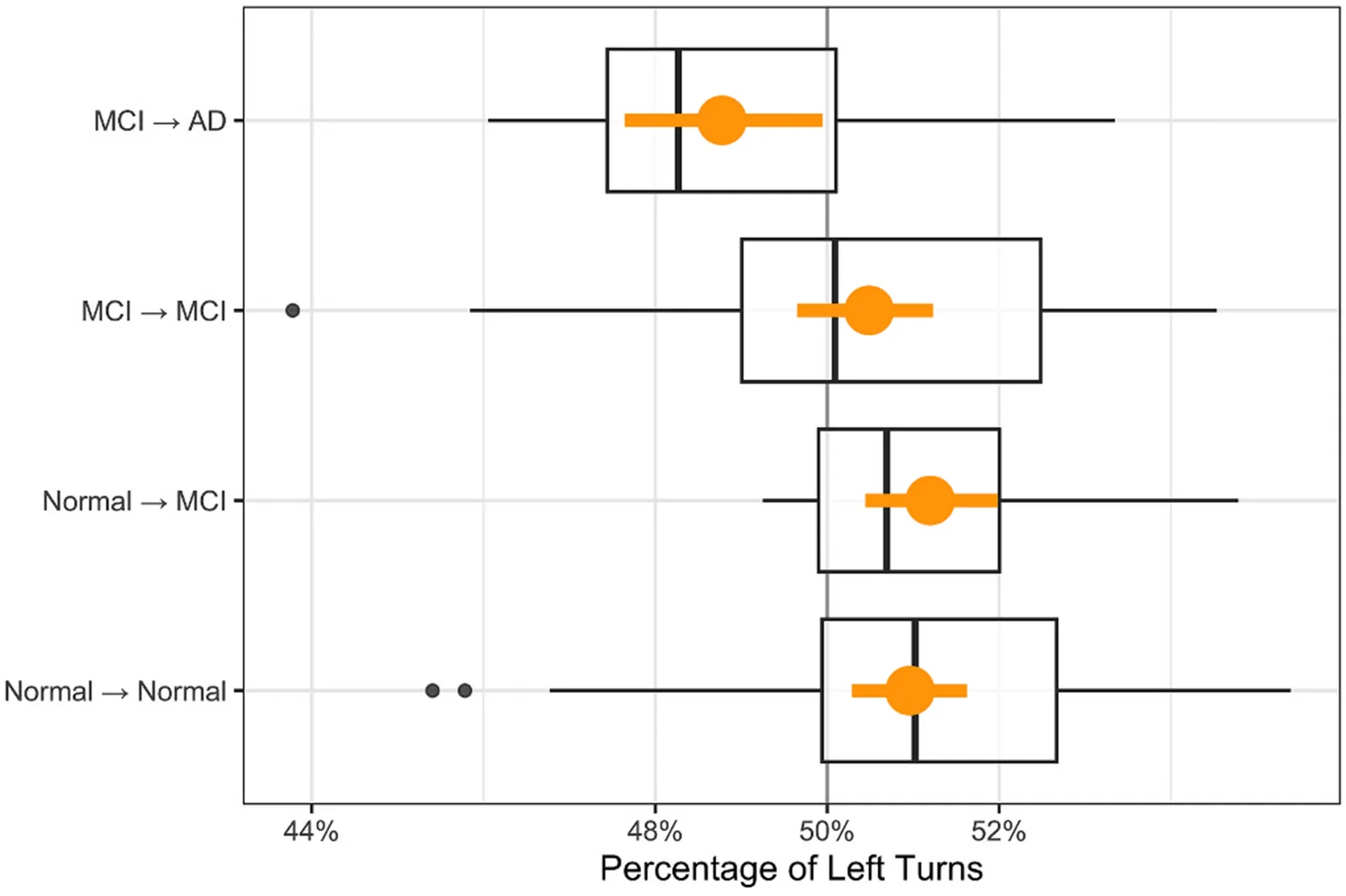
Boxplots of the percentage of left turns for each cognitive prognosis class with a cutoff of 15 months (450 days) from the end of the driving data collection period to the one-year follow-up assessment. The orange intervals are 95 % bootstrap confidence intervals. A decreasing trend is visible in the group medians, represented by the black vertical lines inside the boxes.

**Table 1:** Demographic characteristics and turning endpoints, overall and by cognitive prognosis class.

Characteristic	Value	Overall, n = 106	Normal → Normal, n = 43	Normal → MCI, n = 16	MCI → MCI, n = 36	MCI → AD, n = 11
Age, mean (SD), years		75.3 (6)	74.1 (5.1)	74.2 (6)	76.9 (6.7)	76 (5.9)
Sex	Female	61 (57.5 %)	33 (76.7 %)	8 (50 %)	16 (44.4 %)	4 (36.4 %)
	Male	45 (42.5 %)	10 (23.3 %)	8 (50 %)	20 (55.6 %)	7 (63.6 %)
Race	White	102 (96.2 %)	43 (100 %)	16 (100 %)	33 (91.7 %)	10 (90.9 %)
	Black	3 (2.8 %)	0 (0 %)	0 (0 %)	3 (8.3 %)	0 (0 %)
	American Indian	1 (0.9 %)	0 (0 %)	0 (0 %)	0 (0 %)	1 (9.1 %)
	Asian	0 (0 %)	0 (0 %)	0 (0 %)	0 (0 %)	0 (0 %)
Employment	Retired	82 (77.4 %)	30 (69.8 %)	11 (68.8 %)	33 (91.7 %)	8 (72.7 %)
	Employed full/part time	15 (14.2 %)	8 (18.6 %)	4 (25 %)	2 (5.6 %)	1 (9.1 %)
	Volunteer	8 (7.5 %)	5 (11.6 %)	0 (0 %)	1 (2.8 %)	2 (18.2 %)
	Keeping house	1 (0.9 %)	0 (0 %)	1 (6.2 %)	0 (0 %)	0 (0 %)
Driving Experience, mean (SD), years		58.6 (6.6)	57.9 (6.6)	56.4 (6.3)	60.1 (6.8)	59.4 (6.3)
Weekly Driving Distance, mean (SD)	Miles	85.3 (75.4)	84.7 (78.6)	91.1 (91.3)	81 (71.1)	93.6 (57.8)
	Kilometers	137.2 (121.3)	136.3 (126.5)	146.6 (146.9)	130.3 (114.4)	150.6 (93)
Number of Trips per Participant During the Driving Period, mean (SD)		298.3 (114.5)	285.4 (103.9)	325.4 (149.9)	310.2 (110.6)	270.3 (112.1)
Mileage per Trip, median (IQR)	Miles	5.2 (3.6)	5.5 (3.8)	6.1 (3.3)	4.9 (3.2)	5 (1.8)
	Kilometers	8.3 (5.9)	8.8 (6.2)	9.8 (5.4)	7.8 (5.1)	8 (3)
Number of Turns Detected Per Participant, mean (SD)	Left	1036.8 (505.2)	997.7 (468.3)	1210.6 (658.2)	1044.3 (483.7)	912.7 (467)
	Right	1005.9 (479.7)	949.3 (417)	1162.8 (654.8)	1018 (460.9)	959.5 (492.8)
Percentage of Turns Detected Per Participant, mean (SD)	Left	50.6 % (2.3 %)	51 % (2.3 %)	51.2 % (1.6 %)	50.5 % (2.4 %)	48.8 % (2.1 %)
	Right	49.4 % (2.3 %)	49 % (2.3 %)	48.8 % (1.6 %)	49.5 % (2.4 %)	51.2 % (2.1 %)

NOTE. Percentages may not add to 100 % due to rounding.

**Table 2:** Predicted probabilities (marginal effects) of turning left and corresponding 95 % confidence intervals for each of the four cognitive prognosis classes.

Cognitive Prognosis Class	Predicted Probability of Left Turn	Lower 95 % CI	Upper 95 % CI
Normal → Normal (1)	0.513	0.507	0.520
Normal → MCI (2)	0.508	0.504	0.513
MCI → MCI (3)	0.503	0.498	0.509
MCI → AD (4)	0.498	0.490	0.507

## Data Availability

The data used in this study are sensitive and thus cannot be shared.

## Appendix

**Table A.1:** Sensitivity analysis results for different values of the angle threshold for turn detection. Coefficient estimates and p-values come from the mixed-effects logistic regression model.

Threshold Angle	Total Number of Turns	Number of Left Turns	Number of Right Turns	Coefficient Estimate	P-Value
30	330,333	165,630	164,703	−0.014	0.020
35	311,021	156,223	154,798	−0.016	0.022
40	293,102	147,247	145,855	−0.019	0.007
45	276,885	139,187	137,698	−0.021	0.004
50	262,925	132,230	130,695	−0.019	0.009
55	250,938	126,316	124,622	−0.019	0.010
60	239,526	120,621	118,905	−0.020	0.008
65	228,149	115,274	112,875	−0.019	0.014
70	216,531	109,904	106,627	−0.020	0.011
75	203,946	104,317	99,629	−0.021	0.011
80	189,197	97,622	91,575	−0.019	0.026
85	168,388	87,807	80,581	−0.018	0.052
90	127,017	66,017	61,000	−0.018	0.133
95	84,928	43,959	40,969	−0.008	0.595
100	69,035	35,868	33,167	−0.006	0.718

## References

[R1] Albert MS, DeKosky ST, Dickson D, , 2011. The diagnosis of mild cognitive impairment due to Alzheimer’s disease: recommendations from the national institute on aging-Alzheimer’s association workgroups on diagnostic guidelines for Alzheimer’s disease. Alzheimer’s Dementia 7 (3), 270–279. 10.1016/j.jalz.2011.03.008. PMC3312027 21514249

[R2] Babulal GM, Stout SH, Benzinger TLS, , 2019. A naturalistic study of driving behavior in older adults and preclinical Alzheimer disease: a pilot study. J. Appl. Gerontol. 38 (2), 277–289. 10.1177/0733464817690679. PMC5555816 28380718

[R3] Basulto-Elias G, Hallmark S, Barnwal A, Sharma A, Rizzo M, Merickel J, 2023. Strategy and safety at stop intersections in older adults with mild cognitive impairment and visual decline. Transp. Res. Interdiscip. Perspect. 22 (November), 100939. 10.1016/j.trip.2023.100939. PMC10811907 38283865

[R4] Bayat S, Babulal GM, Schindler SE, , 2021. GPS driving: a digital biomarker for preclinical Alzheimer disease. Alzheimer’s Res. Therapy 13 (1), 115. 10.1186/s13195-021-00852-1. PMC8204509 34127064

[R5] Bayat S, Roe CM, 2022. Driving assessment in preclinical Alzheimer’s disease: progress to date and the path forward. Alzheimer’s Res. Therapy 14 (1), 168. 10.1186/s13195-022-01109-1. PMC9641948 36348410

[R6] Beck LF, Luo F, West BA, 2022. Examining patterns of driving avoidance behaviors among older people using latent class analysis. J. Appl. Gerontol.: Off. J. Southern Gerontol. Soc. 41 (7), 1752–1762. 10.1177/07334648221086953. PMC9233037 35441554

[R7] Braitman KA, Kirley BB, Ferguson S, Chaudhary NK, 2007. Factors leading to older drivers’ intersection crashes: traffic injury prevention. Traffic Inj. Prev. 8 (3), 267–274. 10.1080/15389580701272346. 17710717

[R8] Camilleri L, Whitehead D, 2023. Driving assessment for persons with dementia: how and when? Aging Dis. 14 (3), 621–651. 10.14336/AD.2022.1126. PMC10187703 37191415

[R9] Chang JH, Huang Y, Zhang Y, , 2025. Day-to-day sleep efficiency and driving behaviors in older adults with and without cognitive impairment. Alzheimer’s Dementia: Diagn. Assess. Disease Monit. 17 (3), e70173. 10.1002/dad2.70173. PMC12358007 40827141

[R10] Chang JH, Latona GJ, Rizzo M, Phatak V, Murman DL, 2026. Overnight sleep affects the stability of neuropsychological classification in mild cognitive impairment. Alzheimer’s Dementia 22 (1), e71128. 10.1002/alz.71128. PMC12832612 41582314

[R11] Choi N, Kim J, Lee J, 2025. Unveiling stress triggers for older drivers: scenario development through FGI-based text mining. Transp. Res. Interdiscip. Perspect. 32, 101531. 10.1016/j.trip.2025.101531.

[R12] Di X, Yin Y, Fu Y, , 2023. Detecting mild cognitive impairment and dementia in older adults using naturalistic driving data and interaction-based classification from influence score. Artif. Intell. Med. 138 (April), 102510. 10.1016/j.artmed.2023.102510. 36990588

[R13] Efron B, Tibshirani R, 1993. An Introduction to the Bootstrap. Chapman & Hall.

[R14] FHWA. 1995. Accident Analysis of Older Drivers at Intersections. FHWA-RD-94-021. Federal Highway Administration.

[R15] Howell FJ, Koppel S, Logan DB, 2024. Descriptive and conceptual structure of naturalistic driving study research: a computational literature review. Transp. Res. Interdiscip. Perspect. 27 (September), 101205. 10.1016/j.trip.2024.101205.

[R16] Jak AJ, Bondi MW, Delano-Wood L, , 2009. Quantification of five neuropsychological approaches to defining mild cognitive impairment. Am. J. Geriatr. Psychiatry 17 (5), 368–375. 10.1097/JGP.0b013e31819431d5. PMC2743175 19390294

[R17] Joshi A, Venkatachalapathy A, Merickel J, , 2025. Impact of acute glucose episodes on adherence to speed limits in the naturalistic setting for drivers with diabetes: an application of linear quantile mixed models. Transp. Res. Interdiscip. Perspect. 34 (November), 101633. 10.1016/j.trip.2025.101633.

[R18] Kuznetsova A, Brockhoff PB, Christensen RHB, 2017. lmerTest package: tests in linear mixed effects models. J. Stat. Softw. 82 (December), 1–26. 10.18637/jss.v082.i13.

[R19] Lüdecke D, 2018. ggeffects: tidy data frames of marginal effects from regression models. J. Open Source Software 3 (26), 772. 10.21105/joss.00772.

[R20] Marshall GA, Zoller AS, Lorius N, , 2015. Functional activities questionnaire items that best discriminate and predict progression from clinically normal to mild cognitive impairment. Curr. Alzheimer Res. 12 (5), 493–502. 10.2174/156720501205150526115003 PMC4448081 26017560

[R21] Merickel J, High R, Dawson J, Rizzo M, 2019. Real-world risk exposure in older drivers with cognitive and visual dysfunction. Traffic Inj. Prev. 20 (November), S110–S115. 10.1080/15389588.2019.1688794. PMC7035173 31821019

[R22] Molnar LJ, Charlton JL, Eby DW, , 2013. Self-regulation of driving by older adults: comparison of self-report and objective driving data. Transport. Res. F: Traffic Psychol. Behav. 20. 10.1016/j.trf.2013.05.001.

[R23] Molnar LJ, Eby DW, 2008. The relationship between self-regulation and driving-related abilities in older drivers: an exploratory study. Traffic Inj. Prev. 9, 314–339. 10.1080/15389580801895319. 18696387

[R24] Morris JC, 1993. The clinical dementia rating (CDR). Neurology 43 (11), 2412. 10.1212/WNL.43.11.2412-a. 8232972

[R25] Petersen RC, Smith GE, Waring SC, Ivnik RJ, Tangalos EG, Kokmen E, 1999. Mild cognitive impairment: clinical characterization and outcome. Arch. Neurol. 56 (3), 303–308. 10.1001/archneur.56.3.303. 10190820

[R26] Preusser DF, Williams AF, Ferguson SA, Ulmer RG, Weinstein HB, 1998. Fatal crash risk for older drivers at intersections. Accid. Anal. Prev. 30 (2), 151–159. 10.1016/S0001-4575(97)00090-0. 9450119

[R27] Seacrist T, Douglas EC, Hannan C, Rogers R, Belwadi A, Loeb H, 2020. Near crash characteristics among risky drivers using the SHRP2 naturalistic driving study. J. Saf. Res. 73 (June), 263–269. 10.1016/j.jsr.2020.03.012. 32563402

[R28] Rizzo M, Dawson JD, 2025. “AI in neurology: everything, everywhere, all at once part 1: principles and practice”. Ann. Neurol, ahead of print, June. doi:10.1002/ana.27225. PMC12278199 40536356

[R29] Sifrit KJ, Stutts J, Martell C, Staplin L, 2011. Intersection Crashes Among Drivers in Their 60s, 70s, and 80s. In: DOT HS, 811. National Highway Traffic Safety Administration.

[R30] Williams RA, 2020. Ordinal Independent Variables.” In SAGE Research Methods Foundations. SAGE Publications Ltd.

